# Reconstructing Archaeological Networks with Structural Holes

**DOI:** 10.1007/s10816-017-9335-1

**Published:** 2017-04-21

**Authors:** Viviana Amati, Termeh Shafie, Ulrik Brandes

**Affiliations:** Department of Computer & Information Science, University of Konstanz, 78457 Konstanz, Germany

**Keywords:** Network reconstruction, Structural holes theory, Closure theory, Exponential random graph models

## Abstract

Model-based reconstruction is an approach to infer network structures where they cannot be observed. For archaeological networks, several models based on assumptions concerning distance among sites, site size, or costs and benefits have been proposed to infer missing ties. Since these assumptions are formulated at a dyadic level, they do not provide means to express dependencies among ties and therefore include less plausible network scenarios. In this paper we investigate the use of network models that explicitly incorporate tie dependence. In particular, we consider exponential random graph models, and show how they can be applied to reconstruct networks coherent with Burt's arguments on closure and structural holes (Burt 2001). The approach is illustrated on data from the Middle Bronze Age in the Aegean.

## Introduction

Network approaches have been adopted in many disciplines to analyse relational data. Archaeology is no exception as attested by pioneering studies in the late 60s and 70s (Clarke 1972; Terrell 1977) and recent applications (Knappett 2011; Brughmans 2013). Network perspectives provide archaeology with the potential to analyse contacts between past cultures and communities, so that several phenomena such as migrations (*e.g.* Mills *et al.* 2013), exchange (*e.g.* Gjesfjeld and Phillips 2013), and the emergence of settlement hubs (*e.g.* Broodbank 2002; Malkin 2011) can be better understood. Since archaeological evidence and historical documentation are fragmentary and do not provide enough information to get anywhere near a complete picture of networks in the past, developing methods to reconstruct archaeological networks and infer missing links is crucial.

Several approaches have been used to reconstruct archaeological networks. As suggested by Östborn and Gerding (2014), the main distinction is between procedures that infer links from archaeological data and those that infer links on the basis of assumptions concerning the processes that might have generated past networks. Network reconstructions based on the criterion that co-presence and similarity in archaeological finds between sites are proxy for the existence of ties (*e.g.* Sindbæk 2007; Coward 2010, 2013; Mills *et al.* 2013) are examples of the former approach. Network reconstructions resting on models specified by propositions concerning the generative mechanisms are examples of the second approach.

Various models have been proposed and applied in the archaeological literature. A main distinction is between agent-based models and models that in this paper will be referred to as “tie-based models”. Both models aim at explaining how macro (*i.e* system-level) characteristics of a network emerged from processes taking place at the micro level. However, while agent-based models (Graham 2006; Wurzer *et al.* 2015) rely on explicit assumptions about the behaviours of heterogeneous individuals, the tie-based models hinge on assumption formulated at the tie level, *i.e.* on pairs of nodes (*e.g.* Conolly and Lake 2006; Knappett *et al.* 2008; Terrell 2010). These assumptions are often formulated as tendencies of nodes to form particular ties, and therefore might only implicitly describe the behaviour of the individuals. In this paper, we refer to the tie-based network reconstruction only.

Several deterministic and stochastic tie-based models have been used to reconstruct archaeological networks. Though, in principle, these models could be used to reconstruct networks at various scales, nodes are aggregated units (*e.g.* settlements, communities, islands) in most of the applications (see Section 2 and the references therein). Therefore, in the following, we assume that nodes are settlements, whose existence is proved by the finding of sites, and ties between pairs of nodes represent (the volume of) contacts between them. Sites are characterized by their geographical location and size. The terms “contact” and site “size” are used in a very broad sense here. The former subsumes any type of relation (*e.g.* exchange and material flow), while the latter refers to any attribute describing the importance of each site (*e.g.* the population and the area of extension).

Tie-based models are specified by propositions and assumptions deriving from archaeological theory, which in turn is based on archaeological evidence. The simplest models, *e.g.* maximum distance networks and proximal point analysis, assume that distance between sites play a major role in shaping the network of contacts in the past due to the limitations of travel technology. Gravity models further assume that sites could sustain a different number of contacts according to their sizes. ariadne (Knappett *et al.* 2008) is the most advanced model and aims to reconstruct both the network and the importance of sites. It assumes that ties are both beneficial and costly to sites and plausible reconstructed networks are therefore those that maximize the trade-off between benefits and costs. These networks are referred to as *efficient* networks.

Thus far, all the hypotheses that have been used in archaeology to specify models for network reconstruction are formulated at a dyadic level, *i.e.* on pairs of sites. Specifically, the existence of a tie between any two sites *i* and *j* depends only on (constant or time-dependent) attributes of sites, such as their geographical location or size, and the constraints imposed on them. In network terminology, these models assume dyadic independence, *i.e.* they assume that the probability of observing a tie in the network does not depend on the presence of other ties. Intuitively, the presence of a tie between sites *i* and *j* does not depend on the ties between *i* and any other site, the ties between *j* and any other site, and the ties between any other pairs of sites. This assumption is often unrealistic as, in general, the configuration of a network is also determined by endogenous mechanisms whereby ties come to exist in reaction to the existence of other ties. For instance, if *i*, *j* and *h* are three sites and *i* is exchanging goods with *j* and *j* with *h*, we could suppose that *i* and *h* will start an exchange to directly obtain the respective goods. However, if *i* and *j* are close in space and *h* is far away, it might be more reasonable to assume that sites *i* and *h* will not start an exchange, but rather obtain the respective goods by means of *j*. If that is the case, the resulting network structure would be characterized by a well-developed system of contacts within the close range and the presence of non-redundant outside contacts controlled by broker sites. Networks characterized by this structure will be referred to as *structurally efficient* networks throughout the paper. Since both of the previous assumptions are based on dyadic dependence and the latter also accounts for the interaction between existing ties and distance, earlier models assuming dyadic independence are not able to reproduce structurally efficient networks. These models can therefore only provide a limited number of plausible scenarios for networks in the past.

Several statistical models for the analysis of relational data have been developed in the past three decades (see, *e.g.*, Snijders (2011) and the references therein) to account for dyadic dependence and its interaction with factors exogenous to the network. In this paper, using exponential random graph models (ERGMs) to reconstruct structurally efficient networks in the past is proposed and discussed.

The remainder of the paper is organized as follows. In Section 2 a review of tie-based models used to reconstruct archaeological networks is presented. The definition of structurally efficient networks and the logic behind ERGMs are introduced in Section 3. Section 4 provides an empirical example with results on the reconstruction of networks in the Aegean during the Middle Bronze Age. These results and further developments are discussed in Section 5.

## Review of Models for Network Reconstruction

The main distinction among the tie-based models is between deterministic and stochastic models. According to the former, the existence of a relationship between two sites depends on the fulfilment of certain criteria specified by some assumptions. Consequently, the reconstruction of archaeological networks can be regarded as a deterministic procedure stating which ties exist, leading to one fixed network of contacts. On the contrary, a stochastic model-based approach assumes that a network is the outcome of a probabilistic model which is a synthetic description of the mechanisms that might have generated the network. The specification of the model is determined by a set of assumptions regarding tie formation and a set of parameters. Once the model is fully specified, networks can be sampled from this distribution. Thus, reconstructing archaeological networks means inferring the structure of a network given a set of assumptions regulating the occurrence of ties, and the resulting network is not fixed but subject to the sampling scheme.

Irrespective of the approach used, the reconstruction of archaeological networks requires the formulation of assumptions. A fundamental assumption of all the models we describe in the following is that contacts between sites depend on geographical space, for which distance is usually a proxy (Renfrew 1975; Terrell 1977; Rihll and Wilson 1987; Knappett *et al.* 2008; Evans *et al.* 2009, 2012). The rationale is that distances are determinants of whether passive contacts, which are premises for tie formation, will occur. There are different types of distance particularly relevant to archaeology: *geographical* and *cost-*distance. The former can be a simple (*e.g.* euclidean distance) or more complex (*e.g.* great circle distance) measurement. The latter has a more general definition and accounts for the time and strength required to move from one location to another (White and Surface-Evans 2012; Hill *et al.* 2015 and the reference therein). For instance, uphill movement is slower and more exhausting than downhill movement and, in some circumstances, sea travel might be faster than land travel. Regardless of the definition of distance, the model formulations penalize long-range distances so that contacts between sites close to each other are more likely. Another popular assumption is that contacts depend on the size of sites (Renfrew and Level 1979; Rihll and Wilson 1987; Knappett *et al.* 2008). The size of a site can be understood as resources (*e.g.* population and area of a site) available to a site to maintain the contacts with other sites. It is assumed that the larger the site, the higher its ability to maintain contacts.

The models presented in the following review differ both on the approach taken and as how to model the effect of distance and size of sites on ties. We start by introducing the notations used throughout this paper.

An archaeological network is referred to as a network where the set of nodes N = {1,2,..,*n*} comprises settlements, whose existence is proved by the finding of sites, and ties between pairs of nodes represent (the volume of) contacts between them. Sites are characterized by their geographical location and size. Archaeological networks are represented as graphs, where vertices are sites and edges between nodes are contacts, as well as as an adjacency matrix *x*, *i.e.* as an *n×n* matrix whose generic cell *x*_*ij*_ describes the relationship between *i* and *j*. For binary relations *x*_*ij*_ = 1 if there is a relationship between *i* and *j*, and 0 otherwise. For valued relations *x*_*ij*_ > 0 if there is a relationship between sites *i* and *j*, and 0 otherwise. The set of all the possible network scenarios that can be defined over the set N is denoted by X. Attributes of sites (*e.g.* size and geographical location) are represented by a matrix *v* whose generic element *v*_*ip*_ is the value of site *i* on the characteristic *p*. Finally, the distance between sites is represented as an *n×n* matrix *d* where the generic cell *d*_*ij*_ indicates the (geographical or cost-) distance between sites *i* and *j*.

### Maximum Distance Network

Maximum Distance Network (MDN) is a simple way to construct a network and is based on the hypothesis that binary unweighted networks are only dependent on physical distance: there is a distance (determined by for instance transportation technology) beyond which single journeys between pairs of sites become too difficult to take. Thus, the networks produced capture information about the contacts between sites based on a specified threshold of the distance matrix. These networks can be regarded as inflicting deterrent costs on long single journeys. In the MDN, edges are set as a simple step function of distance which is equal to one if the distance is below a specified threshold, and zero otherwise.

Formally, an undirected tie *x*_*ij*_ between sites *i* and *j* exists if the distance *d*_*ij*_ between the sites is less than a specified model parameter *D*, *i.e.*

1 $$ {x}_{ij}=\left\{\begin{array}{cc}\hfill 1\hfill & \hfill if\kern0.5em {d}_{ij}\le D\hfill \\ {}\hfill 0\hfill & \hfill \begin{array}{cc}\hfill otherwise\hfill & \hfill .\hfill \end{array}\hfill \end{array}\right. $$

In other words, the way in which contact falls off with distance is controlled by *D*. For distances shorter than *D* we assume that journeys are relatively easy to make, and for distances larger than *D*, they are difficult to make. Another way to think of this model is as a quantitative way of defining regional clusters. Since we are connecting sites less than a certain distance *D* apart, only sites within the same cluster are connected to each other by a path.

The equivalence of MDNs in geometric graph theory are *disk-intersection graphs* (Clark *et al.* 1990) in which a disk of radius equal to *D/2* is put around every vertex, and an edge between two vertices appear if the corresponding disks have non-empty intersection. In other words, a disk graph is the intersection graph of a set of disks in the Euclidean plane.

The MDN model is frequently used in archaeology and in particular, on Middle Bronze Age (MBA) networks for varying *D* (Rivers *et al.* 2011, 2013; Evans *et al.* 2012; Rivers *et al.* 2013). In these applications, several points of criticisms are noted by the authors together with some rather intuitive options to overcome these. The MDN model produces simple networks where edges are present or not, and have no weights or direction. This has been considered too simplistic and Rivers *et al.* (2013) emphasised the need to consider travel times for which distance is not always a good proxy. Therefore, introducing a frictional coefficient for land travel in comparison to sea travel is commonly used as an option. Travel times can also be directional, particular for maritime journeys with winds and tides. However, an option to deal with this problem is to expect these trends to cancel each other out over the course of a year so that the distances can be kept non-direction.

The strongest criticism is towards the choice of *D* and the edges that are created based on this choice. If the distance threshold is too small, the network is overpowered by the geographical distances, and if the distance threshold is too large, the whole network becomes over connected, giving rise to more edges than reasonable. Further, while there is no doubt that geography is an important factor for network formation, ease of travel is only one component to take into account.

### Proximal Point Analysis

In MDN, the premise is that communities need to interact but it is just too difficult to travel (as controlled by *D*) while in PPA, the premise is that communities need to travel but it is too difficult to sustain more than a few contacts, as controlled by the parameter *k* defined in the following. The parameter *k* is the number of sites to which a site is connected and it is constant over all the sites. This means that PPA implicitly assumes that sites have the same status, *i.e.* there are no sites to whom all other sites wish to connect to.

In PPA, each site is connected to its *k*-nearest neighbours where by “nearest” is meant geographically closest. For any site *i*, the distances between *i* and all the other *n-1* sites are computed. Let *d*_*i(1)*_ *≤ d*_*i(2)*_ *≤ … ≤ d*_*i(n-1)*_ be the size-ordered sequence of these distances. Then, the existence of a tie between sites *i* and *j* is determined by

2 $$ {x}_{i j}=\left\{\begin{array}{cc}\hfill 1\hfill & \hfill \begin{array}{cc}\hfill if\hfill & \hfill {d}_{i j}\le {d}_{i(k)}\hfill \end{array}\hfill \\ {}\hfill 0\hfill & \hfill \begin{array}{cc}\hfill otherwise\hfill & \hfill .\hfill \end{array}\hfill \end{array}\right. $$

The fact that *j* is a *k*-nearest neighbour of *i* does not imply that *i* belongs to the *k*-neighbourhood of *j*, and therefore the PPA results in a directed network though the directions are usually ignored in the literature. Comparison between the tie definition in (1) and (2) shows that the PPA is a generalisation of MDN. Instead of using a fixed threshold for the existence of a tie like in MDN, PPA considers distance thresholds relative to each site. This results in a different network structure where sites that are separated by large distances from their neighbours will connect nonetheless.

The difference in threshold selection stresses the distinction between the two models. While MDN assumes that sites have the same travel ability (as described by D), in PPA sites have the same capability of sustaining a certain number of connections. Moreover, while MDN is only determined on a geographical base, PPA combine geography and site status together.

Island archipelagos offer appropriate research contexts for the application of PPA because of the clear separation of sites. In particular, early application was to anthropological networks in Oceania where Terrell (1977) used PPA to model the probable behaviour of human populations in Solomon Islands using *k=3*. As the author wrote: “The procedure of drawing three lines out from every single point reflects the assumption that village settlements in the Solomons all enjoy an equal status and that none performs centralized, specialized functions for its surrounding neighbors” (Terrell 1977, p. 37). The final network created shows the most probable directions of inter-island trade, travel, intermarriage, and migration. Terrell (2010) used PPA to explore how variation in the material culture records of Sepic communities may be related to language differences among villages. Here, the expected impact of distance on the weight of contacts among the communities are modelled with a 1st, 2nd and 3rd order PPA for each community. Other archaeological applications of PPA include Rivers *et al.* (2011), Evans *et al.* (2012) and Rivers *et al.* (2013), where it was used to model MBA networks, and Collar (2013), where it was used to analyse the diffusion of religious innovation in the Roman Empire.

The aim of using PPA may be to decide which sites have a more prominent role in regional contacts. This is determined by connectivity or degree centrality where the degree of a site is the number of links that it has with its network neighbours. Usually under PPA, the distribution of sites is uneven so that some sites will start to gather more than their minimum number of ties since they are closer to the other sites in the network. Thus, some sites will appear more connected than other sites, and portrayed as more central in the network. Broodbank (2002) used PPA with *k=3* to simulate Early Bronze Age Cyclades settlement patterns to obtain results of site centrality or remoteness through visual inspection.

Networks built on PPA place a strong emphasis on local geography because most neighbours are relatively close. However, an opposite effect is that isolated sites will interact despite the distances separating them. The networks emerging from PPA will thus lie in a string shape of sites in contrast to MDN networks where sites tend to cluster. Consequently, the resulting networks may often be composed of several connected components linking sites that are not necessary far apart (White 2012). Another downside of PPA is its sensitivity to the emergence of new sites. In particular, the resulting network is highly dependent on the choice of *k*; higher *k* implies denser network (*i.e.* a network with many ties).

In graph theoretical terms, PPA is a variant of *k-nearest neighbour graphs* in which two nodes are connected if the distance between them is among the *k*-th smallest distances from *i* to all other nodes. If the spatial information of the set of nodes (*i.e.* their topological arrangement) is taken into consideration, yet another version of PPA is obtained. These are the *relative neighbourhood graphs* (Toussaint 1980) in which there is a tie between sites *i* and *j* if

$$ \begin{array}{ccc}\kern1em {d}_{ij}\le \max \left\{{d}_{ik},{d}_{jk}\right\},\kern1em & \kern1em  k\ne i, j,\kern1em & \kern1em \forall k\in \mathcal{N}\kern1em \end{array} $$

Thus, the occurrence of a tie between two vertices is determined by taking into account how close two nodes are to each other and the relative distances of every pair to the remaining nodes in the network. The inclusion of a tie is thus governed by determining if any node pairs are relative neighbours or not.

While nearest neighbour techniques measure the absolute distance between the points, the relative neighbourhood concept considers the region of influence for pairs of vertices. The concept also allows the recognition of different levels of neighbourliness by extending or reducing the size of the region of influence. The notion of relative neighbours is used today in many fields to describe morphological properties of empirical networks. This is particularly common in many archaeological contexts where spatial relationships that match symbolic meanings need to be uncovered. For instance, Jiménez and Chapman (2002) examined the spatial distribution of Mexica offerings which can be described as a set of artefacts whose topology is used to interpret their semantic and symbolic values.

### Gravity Model

The hypotheses presented thus far are that sites have a tendency to interact directly with other sites but are limited in doing so, either because it is too difficult to travel far or too difficult to sustain more than a few contacts. Consequently, the networks resulting from MDN and PPA are binary (a tie from *i* to *j* is either present or absent) and determined by the geographical location of the sites or by the number of sustainable connections. However, it is well established in network analysis that ties can depend on node attributes and be characterized by weights describing the strength of the relationship between two nodes. The Gravity Model (GM) and the subsequent ariadne model introduced in the next section allows accounting for these elements.

By analogy with Newton’s law of gravitation, the GM is based on the principle that “an attracting force of interaction between two areas of human activity is created by the population masses of the two areas, and a friction against interaction is caused by the intervening space over which the interaction must take place” (Carrothers 1956, p. 94).

This theory results in the simplest form of the GM, where the number of contacts *x*_*ij*_ between sites *i* and *j* is given by

3 $$ {x}_{i j}={cv}_i{v}_j f\left({d}_{i j}\right) $$

where *c*, usually referred to as *demographic gravitational constant*, is a proportionality factor; *v*_*i*_ and *v*_*j*_ are unspecified attributes of nodes *i* and *j* (such as population size, area, and available resources); and *f*(*d*_*ij*_) : ℝ^+^ → [0, 1] is a cost-distance function (also known as a *deterrence function*). Different functional forms of *f* can be used, given the assumption that the effect of distance on tie existence decreases as distance increases. Inverse power law and exponential decay families are usually good candidates for cost-distance functions. The two families are respectively described by

$$ \begin{array}{ccc}\hfill f\left({d}_{ij}\right)=\frac{1}{{\left(1+\beta {d}_{ij}\right)}^{\gamma}}\hfill & \hfill \mathrm{and}\hfill & \hfill f\left({d}_{ij}\right)=\frac{1}{e^{\alpha {d}_{ij i}}}\hfill \end{array} $$

where *α* and *β* are scaling factors, and *γ* controls the tail weight of the power law distribution. The two families differ in their tail weight, *i.e.* in the number of ties at large distances: for the power law, the tail weight tends to 0 when distance increases more slowly than for the exponential decay.

Given the relation between the GM and the log-linear models for aggregated choices (Sheppard 1978; Anas 1983) or for contingency tables (Willekens 1983), the attracting force *x*_*ij*_ is usually interpreted as the expected volume of contacts between the locations *i* and *j*. The model in (3) implicitly assumes that such volume is symmetric and therefore the network produced by a GM is an undirected valued network. In situations where contacts between two nodes involve some forms of flows, the intensity of the tie from *i* to *j* might differ from that from *j* to *i*. Therefore, more general forms of the GM, yielding directed valued network, have been proposed.

The more generalised form of the GM is the double constrained gravity model (DCGM), particularly used when *x*_*ij*_ is interpreted as the flow from *i* to *j*, and *v*_*i*_ and *v*_*j*_ represent the outflow of the origin and the inflow of the destination respectively. The DCGM accounts for the dependence of the flow from *i* to *j* on both the total flow from *i* to any other sites, and the total flow into *j* from any other sites. This dependence is formulated using the two constraints *v*_*i*_ = ∑_*j*_ *x*_*ij*_ and *v*_*j*_ = ∑_*i*_ *x*_*ij*_, and it is included in the model by replacing the proportionality factor *c* by two sets of balancing factors *A*_*i*_ and *B*_*j*_. This model is given by

$$ {x}_{i j}={A}_i{v}_i{B}_j{v}_j f\left({d}_{i j}\right) $$

Note that the DCGM subsumes the single constrained gravity model (either origin or destination constrained) as a special case. The former can be produced by making one of the balancing factors *A*_*i*_ or *B*_*j*_ equal to one, the latter by making both balancing factors *A*_*i*_ and *B*_*j*_ equal to one (for more details see Wilson (1967)).

The interpretation and the generality of its formula made the GM widely applicable in many disciplines. For instance, in economics the GM is used to explain the volume of trade by country market/production sizes (measured by the Gross Domestic Product). In demography, the GM is applied to describe the volume of migration movements as a function of the country population.

In archaeology, GMs have been frequently applied and recognised as a useful tool in regional analysis since the 70's (Johnson 1977; Conolly and Lake 2006). For instance, Hodder (1974) explained the distribution of Romano-British wares, and the region of influence of some production centres in Southern England using some specifications of the GM. Clarke and Wilson (1983) investigated the sensitivity and the robustness of GMs to the choice of the parameters via simulations. In this direction, Rihll and Wilson (1987) applied the GM to reconstruct the network of contact between known settlements in Ancient Greece dated to the *9* ^*th*^ and *8* ^*th*^ century B.C. By including historical information concerning the importance of poleis in that period, they also validated their model analysing the correspondence between the central position of the settlements and their historical importance. More recently, Wilson (2007) used a GM to analyse the attractiveness and the consumer area of raw material sources in the Vaucluse region of southern France during the Prehistoric period.

### ariadne

The models introduced in the previous sections rely on assumptions that might be too simplistic for some archaeological case studies. For instance, as noted by Knappett *et al.* (2008), the assumption of PPA for reconstructing networks in the Aegean during the Bronze Age is coherent with archaeological evidence only when referring to the early period. Moreover, MDNs, PPA and GMs account only for the effect of site size on contacts without considering the dependence in the opposite direction. The size of sites can indeed affect the tendency of sites to maintain contacts with others, but at the same time contacts might provide resources leading to the growth and the development of the sites themselves. The ariadne model (Knappett *et al.* 2008; Evans *et al.* 2009) was developed in reaction to these limitations.

By analogy with statistical mechanics, Evans *et al.* (2009) assumed that sites in a network can be thought of as interacting particles in an isolated system. Therefore, archaeological networks can be reconstructed using the Gibbs-Boltzmann distribution defined over the set of all the possible networks X on N according to

4 $$ P\left( X= x\right)=\frac{1}{\kappa} \exp \left(- H\left( x, v\right)\right) $$

where *H(x,v)* is the energy of the system, and κ is a normalizing constant assuring that (4) is a proper probability distribution. The energy of the system is determined adopting a cost-benefit approach and plausible networks are those maximizing the trade-off between benefits and costs.

More specifically, for each site costs and benefits depend on the availability of local resources used to sustain the population, the population itself and the distance between sites. The benefit of the local resources is proportional to the rate of growth of the population when the system is isolated (a reasonable assumption for an island setting). It follows that the benefit of local resources for site *i* can be evaluated using a logistic population model (Verhulst 1838)

$$ \begin{array}{cc}\hfill {R}_i=\alpha {v}_i\left(1-\frac{v_i}{k_i}\right)\hfill & \hfill, \hfill \end{array} $$

where *k*_*i*_ is the fixed assigned carrying capacity (*i.e.* the sustainable population), *v*_*i*_ is the actual population of site *i* and *α* is a proportionality factor. *R*_*i*_ is positive (negative) when the local resources are (not) sufficient to maintain the current population, a situation that happens when *v*_*i*_ *< (>) k*_*i*_. Since local resources are limited, each site has to face a cost

$$ {C}_i=\gamma {v}_i $$

associated with the maintenance of the population. The parameter γ quantifies the cost for each unit of the population.

When *R*_*i*_ is negative, then sites need to interact with other sites to sustain their current population. The benefit of exchange from site *i* to site *j* is quantified using the gravitational law

$$ {E}_{i j}=\beta {v}_i{v}_j f\left({d}_{i j}/{d}_M\right){x}_{i j} $$

where *f(d*_*ij*_ */d*_*M*_ *)* is the cost-distance function, with *d*_*M*_ the maximum daily travel distance, *x*_*ij*_ (0 < *x*_*ij*_ < 1, ∑_*j*_ *x*_*ij*_ = 1) is the volume of contacts from site *i* to site *j*, and *β* is a parameter playing the role of the gravitational constant. When *β* is positive, contacts with large sites located nearby are more rewarding. Following the approach in the analysis of economic networks, contact ties provide sites with benefits and, at the same time, entail costs to be created and maintained (Jackson 2010; Goyal 2012). The cost for a site *i* to sustain the tie towards site *j* is represented by

$$ {T}_{i j}=\delta {v}_i{x}_{i j}, $$

where *δ* is a parameter indicating the cost for each unit of the population and volume of contacts.

Given the set of costs and benefits for each site, the ariadne model assigns to each possible configuration of site size and ties variables the “cost-benefit” function

$$ H\left( x, v\right)=-\sum_i\left({R}_i-{C}_i\right)-\sum_{i j}\left({E}_{i j}-{T}_{i j}\right), $$

where the parameters *α* and *γ* are assumed to be constant over all the sites, while the parameters *β* and *δ* are assumed to be constant over all the pairs of sites.

Different values of *H(x,v)* correspond to different network configurations. To obtain plausible networks for the archaeological context, Knappett *et al.* (2008) suggested to determine networks characterized by the highest trade-off between the benefits and the costs, *i.e.* networks minimizing *H(x,v)*. In other words, given the assumptions of the ariadne model, archaeological networks are reconstructed using the most likely networks according to the distribution in (4). However, the cost-benefit function can have several local minima, and the minimum of *H(x,v)* cannot be computed in an analytical way. Therefore, Knappett *et al.* (2008) suggested to use a stochastic approach based on simulated annealing to minimize *H(x,v)*. Simulated annealing (Kirkpatrick *et al.* 1983; Robert and Casella 2004; Burke and Kendall 2005) is a probabilistic procedure to approximate the global optimum of a cost function that might have several local minima. At each step of the algorithm a change to either the network or the site size can take place. Given the current state of the network, an element of the vector *v* or a cell of the matrix *x* is randomly selected and a new value is proposed. If the new value decreases the “cost-benefit” function, the new value is kept and the proposed network is accepted as the next state of the chain. Otherwise the proposed network is accepted with a probability depending on the difference between the “cost-benefit” functions of the current and proposed networks, and a rescaling parameter (defined as the inverse of temperature) avoiding the trapping attraction of the local minima. If there are too many consecutive changes the rescaling parameter is increased. These steps are repeated until the proposed networks are rarely accepted and the current state of the chain is returned. The procedure results in directed weighted networks corresponding to the local minima of *H(x,v)* and considered as approaching the optimal solution.

Intuitively, the optimal network is approached by moving from one network to a slightly different network (where a new value for either a tie or a site size is proposed) which is quasi-efficient or more efficient than the original one. Given the propositions of the ariadne model, a possible interpretation of the algorithm is that the efficient network is the result of a series of (tie or size) changes made by sites to minimize the trade-off between the costs and benefits of the entire system.

Compared to the previous models ariadne aims to reconstruct past networks accounting for the interdependence between strength of contacts and size of sites. While the reconstruction of the network is based on the usual variables (*i.e.* distance and size), the distinctive element of ariadne is the assumption that networks in the past were shaped by costs and benefits entailed by ties and populations. Moreover, ariadne enables us to reconstruct missing links and the sizes of sites at the same time, thereby explaining in more details why certain sites become more important than others.

Applications of this model can be found, for instance, in Knappett *et al.* (2011) and Evans *et al.* (2012).

## Structurally Efficient Networks

The models presented in the previous section reconstruct archaeological networks on a dyadic basis. In particular, they assume that pairwise site characteristics (*e.g.* size and location) and the constraints that are imposed on them are the only determinants of the existence of ties. However, the shape of a network is also determined by its self-organizing tendencies, *i.e.* endogenous mechanisms modelling the dependence of ties on other ties and their interaction with exogenous factors such as site attributes. In general, it cannot be assumed that site *i*’s relation with site *j* is completely independent of its relation with site *k* even after controlling for site attributes. Accounting for tie dependence opens up the development of more accurate models for network reconstruction and the possibility of investigating alternative scenarios.

The ariadne model postulates that networks in the past were *efficient* in the sense that ties with a positive trade-off between costs and benefits were more likely to exist. Since costs and benefits are computed at a dyadic level, networks generated using the ariadne model will hereafter be referred to as *dyadically efficient* networks.

Here, we extend the concept of dyadic efficiency to higher order, “structural,” efficiency. In particular, we assume that archaeological networks were *structurally efficient* according to the reconciliation between closure theory and structural holes theory described by Burt (2001).

In the analysis of social capital, ties are usually considered as channels allowing flows of information or goods between nodes. Coleman’s closure theory (Coleman 1988, 1990) claims that social capital is the result of a network of strongly connected nodes. Since the strong connections facilitate trust and the sharing of knowledge, well-connected nodes have more opportunities to acquire information and produce social capital. On the contrary, Burt’s argument of structural holes (Burt 1992) states that social capital is a “function of brokerage opportunities” (Burt 2001, p.34), *i.e.* it is “created by a network in which people can broker connection between otherwise disconnected segments” (Burt 2001, p.31). The absence of ties between these network segments generates structural holes.

Burt’s theory is based on Granovetter’s (1973) strength of weak ties argument, according to which strong ties (*i.e.* frequent ties) with nodes that are themselves strongly related provide less valuable information than weak ties with nodes that are not connected with each other. Indeed, strong ties within groups expose nodes to the repetition of the same information, while weak ties between groups yield the acquisition of more diverse information. Therefore, nodes that are tied to nodes belonging to different groups may have the opportunity to get richer information. These nodes are referred to as *brokers*. It follows that the structure of the network plays an important role in the diffusion of information and some structures may perform better than others.

According to Burt’s theory, a structurally efficient network is characterized by the presence of strongly connected groups and brokers mediating the flow of information and filling the holes between these groups. This structure reduces the redundancy of information and allows the flow of information between parts of the network that are weakly connected.

**Fig. 1:**
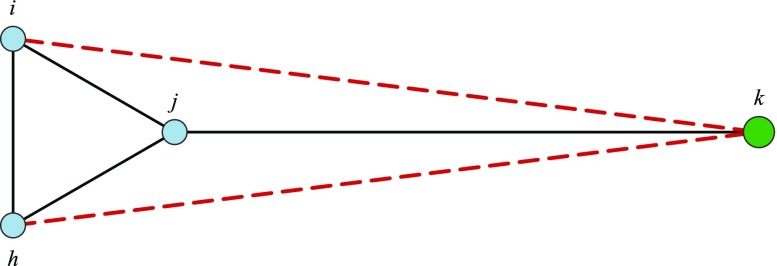
A contact network defined over four sites located in some geographical space. Nodes having the same colour belong to the same neighbourhood. Solid lines represent the ties between the sites, dotted lines represent redundant ties

Burt’s argument combines network closure and structural holes theories and can be applied to the reconstruction of archaeological networks. A structurally efficient network consists of a well-developed system of contacts within the close range of sites and the presence of non-redundant outside contacts controlled by broker sites. The rationale behind this definition is explained here by way of an example. Let *i*, *j*, *h* and *k* be four sites. Fig. 1 shows the location of these sites in the geographical space and the ties among them (solid line). According to network closure, sites in contact with a third site might be more likely to be in contact themselves. Therefore, *i* and *h* may start a contact tie with *k* as well (dotted line). However, the benefits of these two ties are likely to be lower than the corresponding costs. Since *k* is in a different neighbourhood, the effort of maintaining a contact with this site would not be negligible. Moreover, the existence of the ties between *j* and all the other sites already assures that the two neighbourhoods are in contact. Based on these considerations, multiple ties connecting the two neighbourhoods would not be created and *j* would continue playing the role of a broker filling the hole between the neighbourhoods. Multiple ties connecting two neighbourhoods will hereafter be referred to as *redundant ties* and their percentage in a network will be used as a simple way to quantify the redundancy of contacts in a network. The higher the percentage, the less structurally efficient the network.

The example shows that the existence of the tie between *i* and *k* depends on the existence of the ties between *j* and *i*, and *j* and *k* (similarly for the tie between *h* and *k*). This suggests that the reconstruction of structurally efficient networks requires the development of models capable of dealing with tie dependence. Several statistical models for the analysis of network data have been specifically developed to account for tie dependence. Thus, they might represent a useful tool to reproduce archaeological networks.

In the following, we focus on Exponential Random Graph Models (ERGMs), a family of models used to describe the structure of an observed network. While Brughmans *et al.* (2014, 2015) applied ERGMs to model visibility networks in the past, here we discuss their use to reconstruct structurally efficient networks. Although a recent extension allows modelling some forms of valued networks (Krivitsky 2012), and ERGMs are defined for both directed and undirected ties, we consider only ERGMs for undirected binary networks to illustrate their potential to infer missing links in past networks.

### Exponential Random Graph Models

Exponential Random Graph Models (ERGMs) (Wasserman and Pattison 1996; Robins *et al.* 2007; Lusher *et al.* 2013) are stochastic models for describing, modelling and testing hypotheses concerning the structure of one single observation of a complete network. They assume that an observed network results from the combination of several small blocks of ties, referred to as *local configurations*, representing the micro-mechanisms that might have generated the observed network. These micro-mechanisms may depend on both endogenous effects (*e.g.* triangles), related to the self-organizing tendencies of the network *x*, and exogenous effects, concerning the characteristics *v* of the nodes (*e.g.* size) or the attributes *w* of pairs of nodes (*e.g.* distance).

Formally, ERGMs are probability distributions over the set X of all the possible networks defined over N and having the form

5 $$ P\left( X= x\right)=\frac{1}{\kappa} \exp \left(\sum_{k=1}^K{\theta}_k{g}_k\left( x, v, w\right)\right) $$

The distribution (5) suggests that the probability of observing a network *x* depends on a linear combination of statistics *g*_*k*_ *(x,v,w)*, counting the number of local configurations of type *k*, and parameters *θ*_*k*_ measuring the importance of each local configuration in shaping the network *x*. A large and positive (negative) value of *θ*_*k*_ implies that a higher (lower) number of configurations of type *k* is observed in *x* than expected by chance, thereby providing evidence for (against) the corresponding social process. The normalizing constant $\kappa =\sum_{x\in \mathcal{X}} \exp \left(\sum_{k=1}^K{\theta}_k{g}_k\left( x, v, w\right)\right)$ ensures that (5) is a proper probability distribution. We can observe that the model in (5) has the same mathematical form of the ariadne model, but here the energy of the system is represented by the linear combination of the statistics and the parameters.

Tie dependence assumptions define the set of admissible statistics specifying an ERGM. Theories concerning the local mechanisms regulating the creation and the maintenance of ties determine which statistics *g*_*k*_ *(x,v,w)* should be chosen, among those admissible, to specify the model. The number of triangles is an example of statistic. This statistic is used to model closure, *i.e.* the mechanism described by the fact that the contact of my contact is also my contact. As described in the next section, it is through the statistics that the assumptions characterizing the previous models, and many other assumptions, can be incorporated in an ERGM to reconstruct archaeological networks.

Intuitively, an ERGM is a probability distribution for the network variable *X.* This probability can also be interpreted as a logistic regression model. In fact, the network random variable *X* can be thought of as a collection of the tie random variables *X*_*ij*_ ,  *i* , *j*∈ N, taking value 1 if the tie between *i* and *j* is present and 0 otherwise. Given an ERGM, the logarithm of the ratio between the probabilities of a tie being present and of a tie being absent, conditional on all the other ties in the network, can be expressed in the following form:

6 $$ \log \frac{P\left({X}_{ij}=1|{X}_{- ij},\theta \right)}{P\left({X}_{ij}=0|{X}_{- ij},\theta \right)}={\theta}_1\left({g}_1\left({x}^{+}, v, w\right)-{g}_1\Big({x}^{-}, v, w\Big)\right)+\dots +{\theta}_k\left({g}_k\left({x}^{+}, v, w\right)-{g}_k\Big({x}^{-}, v, w\Big)\right) $$

where *X*_−*ij*_ is the set of all the tie random variables other than *X*_*ij*_, and *x* ^+^ and *x* ^−^ are the networks where tie between *i* and *j* is present and absent respectively.

Equation (6) suggests that an alternative formulation of the ERGMs is similar to a logistic regression model where the explanatory variables are differences in the count of the local configuration between the networks *x* ^+^ and *x* ^−^. Consequently, we can interpret the ERGM parameters in the same way we interpret the parameters of a logistic regression model. For instance, if *g*_*k*_(*x*, *v*, *w*) is the number of triangles, the greater the value of *θ*_*k*_, the greater the probability of the presence of a tie leading to higher values of triangles, everything else being equal in the network. A “social” interpretation of this is that nodes have a tendency/preference for forming ties that close two-paths so that the contact of my contact is also my contact. In other words, there is evidence for closure.

Given a specification of an ERGM and values for the parameters *θ*_*k*_, it is possible to sample random networks from (5) using Markov Chain Monte Carlo (MCMC) methods (Gilks *et al.* 1996; Robert and Casella 2004). The main idea of MCMC methods is to simulate the steps of a Markov Chain of networks whose stationary distribution is (5). At each step, one dyad is randomly selected with uniform probability and the tie between them is removed or added according to whether they were already connected or not. If the probability of the network with one changed tie (proposed network) is higher than the probability of the current network, then the proposed network is accepted as the next state of the chain. Otherwise, the proposed network is accepted with a probability depending on how much more likely the current network is with respect to the proposed network. When the number of steps is big enough and the simulated chain has reached the stationary distribution, networks from this distribution can be returned. A “social” interpretation of this algorithm is that the simulated networks are generated by a series of tie changes determined by the tendency/preference of nodes for forming ties according to particular mechanisms. This series of changes is represented by the steps of the algorithm.

It follows that when archaeological assumptions can be translated into network configurations, an ERGM can be specified and networks whose structure is coherent with archaeological evidence can be generated. Moreover, multiple network scenarios can be simulated and compared when, due to the uncertainty deriving from fragmentary data, there are competing hypotheses compatible with archaeological knowledge.

### ERGM Specification for Contact Networks

Several statistics must be included in ERGMs to reproduce networks coherent with the assumptions defining the previous models and to account for structural holes and network closure theories. These statistics, along with the corresponding local configurations and parameter interpretations, are described in Table 1. This table illustrates the correspondence between the local configurations and the propositions (first two columns), the mathematical formulation of the statistics according to the notation introduced in the previous paragraph (third column) and the parameter interpretation. We consider here the case of undirected networks. Equivalent statistics can be defined for directed networks (Robins 2011).

**Table 1:**
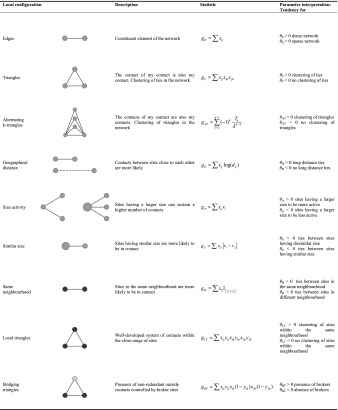
ERGM specification for the reconstruction of archaeological contact networks. Each row provides a description of the local configurations along with the theories they represent, the mathematical formulation of the corresponding statistics and the parameter interpretation. *x* represents the adjacency matrix of the network (*x*_*ij*_ = 1 if sites *i* and *j* are in contact; 0 otherwise), *v* the size of sites, *d* the distance (*d*_*ij*_ is the distance between sites *i* and *j*) and *y* the neighbourhood (*y*_*ij*_ = 1 if sites *i* and *j* are neighbours; 0 otherwise). The symbol ${\mathbb{I}}_{\left\{{y}_i={y}_j\right\}}$ is an indicator function taking value 1 if sites *i* and *j* are in the same neighbourhood and 0 otherwise

Ties are the constituent elements of a network. Networks can be dense (*i.e.* having many ties) or sparse (*i.e.* having few ties). To control for the number of ties in an archaeological network, the statistics *g*_*E*_ counting the number of edges is included in the ERGM. Positive (negative) values of the corresponding parameter *θ*_*E*_ lead to dense (sparse) networks. The statistic *g*_*E*_ must always be included in ERGMs since all the other statistics are combinations of ties and attributes.

The idea that characteristics of nodes can influence the existence of ties has a long tradition in ERGMs and therefore several local configurations have been defined to account for the dependence of ties on node attributes.

One of the assumptions common to all the models summarized in Section 2 is that contacts between sites close to each other are more likely. This idea underlines the importance of geographical space in both formation (*e.g.* MDNs and GMs) and maintenance (*e.g.* PPA, RNG and ariadne models) of ties. To define the corresponding statistic *g*_*D*_, we observe that if sites had a tendency to form geographically bound ties, the sum of the distances between connected sites would be small. Otherwise, we would expect this sum to be quite large. Therefore, a suitable statistic for this assumption is the sum of the ties weighted by the distance. Weighting ties by the logarithm of the distance corresponds to assume that the likelihood of a tie between two sites falls off according to the inverse power law function (Daraganova *et al.* 2012). Positive (negative) values of the parameter *θ*_*D*_ indicate that long-distance ties are more (less) likely than short-distance ties.

The assumptions that “larger sites can sustain a higher number of contacts” and “sites prefer to be in contact with sites with a similar size” are included in the ERGM specification via the size activity and the similar size configurations. If bigger sites could sustain a higher number of relations, then the count of ties for the larger sites would be higher than that for the smaller sites. Therefore, a suitable statistic *g*_*A*_ controlling for the activity of sites having a certain characteristic is defined as a weighted count of ties, where the weights are the size of the sites. Positive (negative) values of the parameter *θ*_*A*_ suggest that bigger sites tend to have more (less) contacts than smaller sites. The same argument applies to the assumption concerning the similarity with respect to the site size. The corresponding statistic *g*_*S*_ is defined as a weighted count of ties where the weights are the absolute differences between the sizes of the sites. Positive (negative) values of the parameter *θ*_S_ indicate that ties between sites with a large difference between site sizes are more (less) likely.

Closure and structural holes theories are modelled via local and bridging triangle configurations (Table 1) respectively. The local triangle statistic *g*_*LT*_ counts the number of triangles within neighbourhoods, whereas the bridging triangle statistic *g*_*BT*_ counts the number of triangles between two different neighbourhoods, such that two nodes belong to the same neighbourhood and one belongs to a different one. The ties between the two nodes in the same neighbourhood and the node in the other neighbourhood are what we have defined as redundant ties. The rationale behind the definition of these statistics is that, if networks in the past were efficient, than the number of triangles within the neighbourhood would be high and the number of triangles between two different neighbours would be small. Positive (negative) values of the corresponding parameters *θ*_*LT*_ and *θ*_*BT*_ suggest a tendency towards (against) local and bridging triangles. Therefore, structurally efficient networks result from models with positive *θ*_*LT*_ and negative *θ*_*BT*_.

Local and bridging triangles are defined as interactions between triangles and the propensity of sites to form ties between sites in the same neighbourhood. Consequently, we should control for the tendency of ties to cluster into triangular structures and form homophilous ties with respect to the neighbourhood. The term “controlling for” means that we need to check if there are other competing mechanisms that might have generated a similar network outcome. For instance, the tendency of sites to connect to the contacts of their contacts (closure) might also lead to a high number of local and bridging triangles. Therefore, the corresponding statistics *g*_*T*_ counting the number of triangles in the network should be included in the model. The tendency towards closure might be stronger according to the number of sites that are intermediaries. This is modelled by the alternating-k-triangles statistic *g*_*AT*_. Positive (negative) values of the corresponding parameters *θ*_*T*_ and *θ*_*AT*_ suggest a tendency towards closure and the overlapping of triangles respectively.

Similarly, the tendency of sites to connect to sites within the neighbourhood might also lead to a high number of local triangles. Therefore, the statistics *g*_*H*_ counting the number of ties within the same neighbourhood is included in the model to control for the tendency of sites to form ties within a neighbourhood. Positive (negative) values of the corresponding parameter *θ*_*H*_ indicate a tendency towards (against) the formation of ties between sites belonging to the same neighbourhood.

Given this specification of the model, simulations of archaeological networks can be conducted using the package *ergm* (Hunter *et al.* 2008; Handcock *et al.* 2014) implemented in R. While the local triangle statistic is already defined in the package, the bridging triangles statistic was added using the package *ergm.userterms* (Hunter *et al.* 2013; Handcock *et al.* 2013).

## Application: Middle Bronze Age in the Aegean

**Fig. 2:**
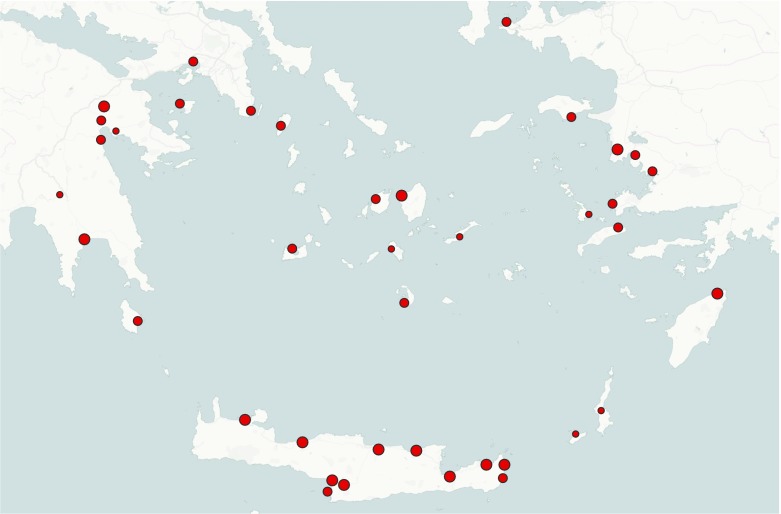
Important sites for the Middle Bronze Age in the Aegean. Size of nodes represents the size of sites

By way of example, data collected by Knappett *et al.* (2011) are used to reconstruct structurally efficient networks during the middle bronze age (MBA) in the Aegean. The data set includes 39 major sites along with their geographical coordinates, cost-distance and size of local resources (Fig. 2). Cost-distance is computed as a linear combination of land and sea distance weighted by frictional coefficients. We hereafter use the values 1 and 2 for the frictional coefficients of sea and land travel respectively, thereby assuming that travelling by land is slower and more costly than travelling by sea. The size of sites is described by an ordinal categorical variable with categories: “1 = small”, “2 = medium” and “3 = large”. Data and their description can be downloaded from http://figshare.com/articles/Thirty_Nine_Aegean_Minoan_Sites/97395.

**Fig. 3:**
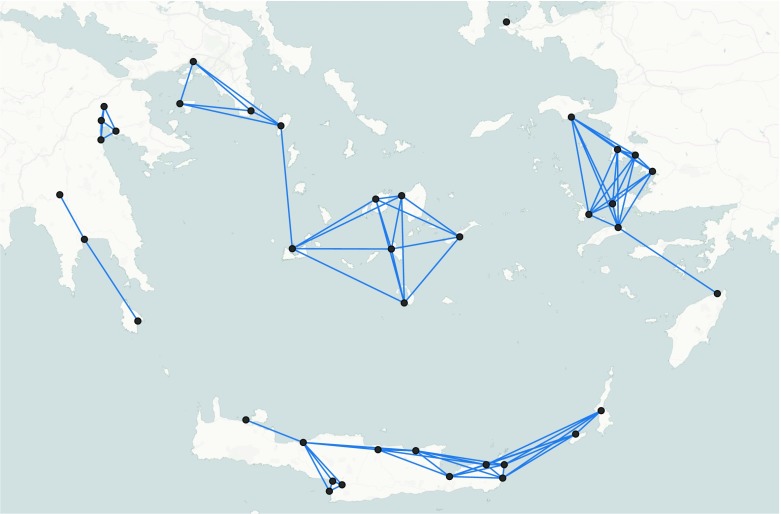
Neighbourhood of sites in the Middle Bronze Age in the Aegean. A tie indicates that two sites are neighbours. Two sites are neighbours if the distance between them is less than 110 kilometres

The definition of neighbourhoods can be based on several criteria. One simple specification depends on the location of sites so that sites lying in the same geographical region are part of the same neighbourhood. Another specification is based on the maximum daily travel distance *d*_*M*_ so that two sites are neighbours if the distance between them is less than *d*_*M*_. The choice between these two criteria is motivated by the same reasons leading to the decision between using geographical distance or cost-distance. In fact, lying in the same geographical region does not always facilitate the creation and the maintenance of ties. In the following, neighbourhoods are defined using the maximum daily travel distance, which for the MBA is *d*_*M*_ = 110 kilometres (Knappett *et al.* 2011). Neighbourhoods of sites are represented by a matrix *y*, with *y*_*ij*_ = 1 if *i* and *j* are neighbours and 0 otherwise, and visualised in Fig. 3. The presence of a tie indicates that two sites are neighbours, *i.e.* the cost-distance between them is lower than 110 kilometres.

Like any ERGM, a model including the statistics in Table 1 must be fully specified to simulate structurally efficient networks. We propose to establish appropriate magnitudes by first estimating parameters of the specified ERGM on a network obtained from one of the models described in Section 2 and then tuning these estimated parameters according to the underlying assumptions.

Note, however, that not all the networks generated from the previous models are suitable for this approach. In fact, the outcome of the MDN based on maximum travelling distance corresponds to the neighbourhood structure depicted in Fig. 3. Hence, the statistics related to the number of edges and the same neighbourhood are collinear and the specified ERGM cannot be estimated. When statistics are collinear different combinations of parameter values could represent the same data pattern (*i.e.* the same values of the statistics) and therefore the estimation algorithm will not converge and return high standard errors. Problems arise also when considering the outcome of the PPA. In this case, the model estimates are inflated because of complete separation (*i.e.* there is a threshold value of the exponent of the ERGMs above which ties are always predicted to be present) no matter what the value of *k* is. Networks produced by (simple) GMs and the ariadne model cannot be used directly since they are weighted and those deriving from the ariadne model are also directed. These networks can be transformed into undirected binary networks by keeping only those ties whose values exceed a certain threshold and ignoring their direction. While the estimation of the ERGM on the binary outcome of the GM is affected by complete separation and therefore cannot be used in this specific case, considering a binary undirected outcome of the ariadne model provides valid estimates. Thus, in this application, the values of the parameters are chosen and tuned based on networks generated with the ariadne model.

**Fig. 4:**
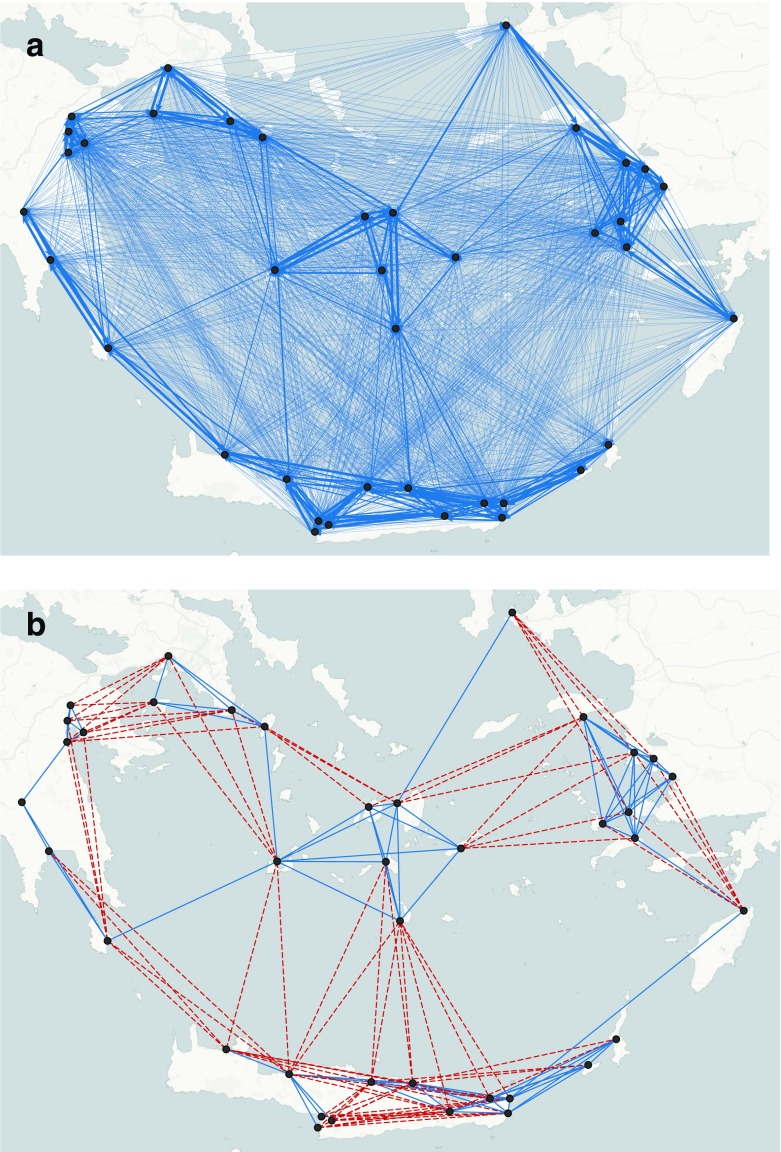
a) Outcome of the ariadne model with parameter values *α* = 4, *β* = 1, *γ* = 0.1, *δ* = 2 and *d*_*M*_ = 110 kilometres. The thickness of the lines represents the weights of ties (the thicker the line, the higher the volume of interaction) b) The undirected binary network computed from the ariadne model in b using a threshold equal to the mean of the weights (= 0.0256). 48% of ties are redundant (dotted lines)

Fig. 4b shows an example of an undirected binary network generated by the ariadne model with parameter values *α* = 4, *β* = 1, *γ* = 0.1, *δ* = 2 and *d*_*M*_ = 110 kilometres (Knappett *et al.* 2011) and a threshold for the presence/absence of ties equal to the mean of the weights of all the ties. The original weighted network is shown in Fig. 4a. Given the binary network in Fig. 4b, we estimated the ERGM specified by the statistics described in Table 1 using the *ergm* package (Hunter *et al.* 2008; Handcock *et al.* 2014) in R. Table 2 illustrates the corresponding estimates. Only the parameters concerning the assumptions leading to the ariadne model turned out to be significant. The parameter related to the distance effect is, indeed, negative implying that there is a tendency towards the formation of ties between sites in a close range. Moreover, the parameters related to the size of the sites are positive suggesting that medium and large sites sustain a higher number of relations. The parameter of the alternating-k-triangles is also significant and positive providing evidence for the tendency to share several contact sites. The model estimates show that there is no evidence for structural holes and closure theories. Although the parameters are not significantly different from zero, they are both positive, thereby implying evidence for closure theory but against structural holes theory. The interpretation of the parameters explains the remarkable presence of redundant ties in the binary undirected network: 48% (78 out of 161) of ties are in fact redundant (Fig. 4b). Note that the percentage of redundant ties can be reduced increasing the threshold used for creating the binary network. However, the resulting network will be composed of several disconnected components and therefore structurally inefficient.

**Table 2:** ERGM estimates (*θ*) and corresponding standard errors (s.e.(*θ*)) for the undirected binary network represented in Fig. 4b

	*θ*	s.e.(*θ*)
Edges	9.324	2.735***
Triangles	- 0.494	0.300***
Alternating-k-triangles (λ = 1.5)	0.988	0.272***
Local triangles	0.107	0.395***
Bridging triangles	0.018	0.137***
Distance (logarithm)	-2.784	0.484***
Size activity (medium)	0.716	0.377**
Size activity (large)	0.924	0.349***
Similar size (diff=1)	-0.239	0.287***
Similar size (diff=2)	0.312	0.569***
Same Neighbourhood	0.478	1.159***

*** p < 0.001, ** p < 0.01, * p < 0.05, ^•^ p < 0.1

**Fig. 5:**
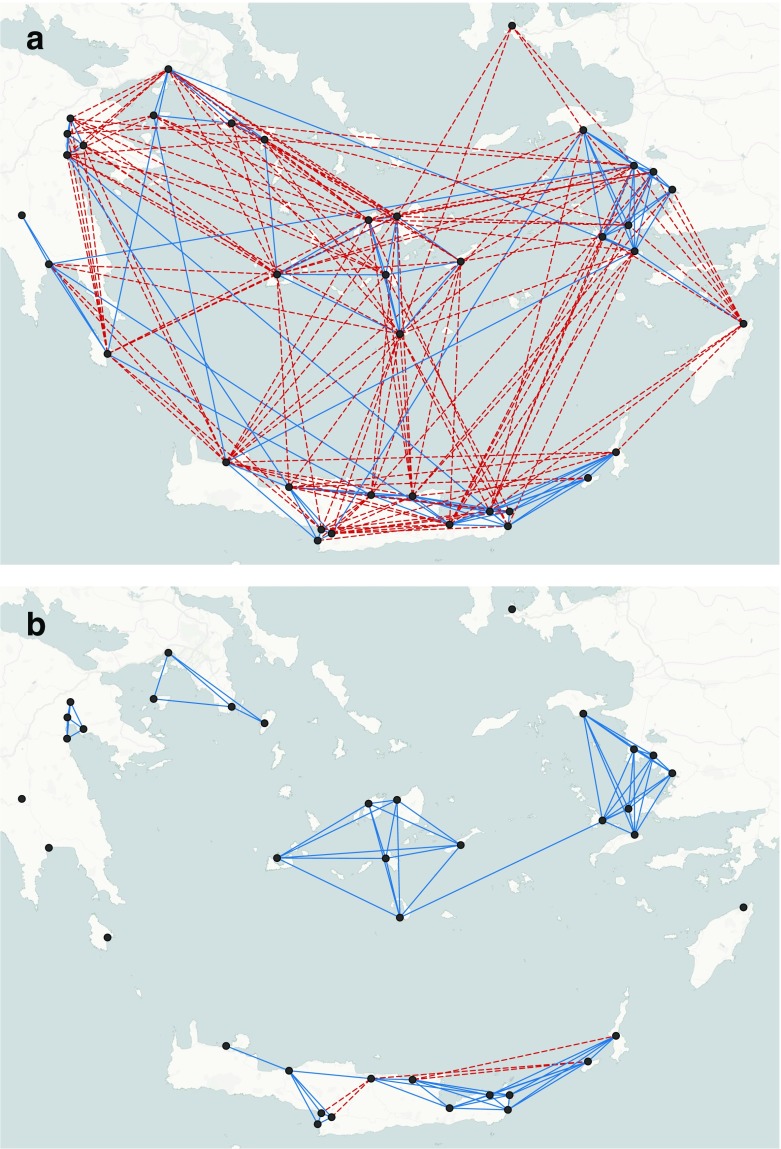
Effect of the distance parameter: a) *θ*_*D*_ = -2.6; redundant ties 60%. b) *θ*_*D*_ =-2.9; redundant ties 6% (dotted lines represent redundant ties)

Tuning the parameters in Table 2 enables us to reconstruct possible scenarios based on Burt’s theory. Fig. 5 shows the effect of the distance parameter *θ*_*D*_. Increasing *θ*_*D*_ yields dense networks characterized by long distance ties and a high percentage (63%) of redundant ties (Fig. 5a), whereas decreasing *θ*_*D*_ leads to disconnected networks with a few (6%) redundant ties (Fig. 5b). None of these networks is structurally efficient suggesting that distance per se cannot yield scenarios where broker sites bridge well-connected regions of a network. Furthermore, since the definition of neighbourhoods is based on the maximum daily travel distance, the number of redundant ties is simply reduced because of the tendency of ties to form in a close range rather than the presence of broker sites.

**Fig. 6:**
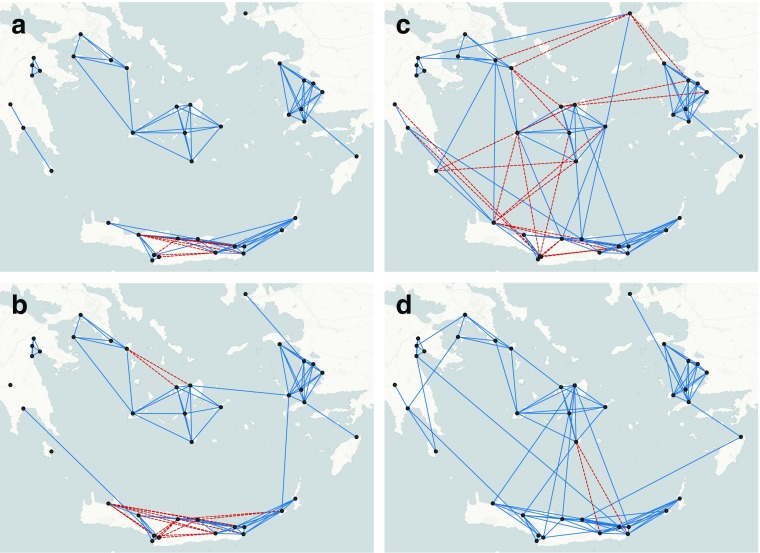
Applying Burt’s theory. Varying the parameter values of: a) local and bridging triangles: *θ*_*LT*_ = 0.5, *θ*_*BT*_ = -0.3; redundant ties 9%; b) local and bridging triangles: *θ*_*LT*_ = 0.3, *θ*_*BT*_ = -0.1; redundant ties 19%; c) triangles, alternating-k-triangles, local and bridging triangles, distance and same neighbourhood: *θ*_*T*_ = -0.2, *θ*_*AT*_ = 1.0, *θ*_*LT*_ = 0.9, *θ*_*BT*_ =-1.8, *θ*_*D*_ = -2.6, *θ*_*S*_ = 0.9; redundant ties 20%. d) triangles, alternating-k-triangles, local and bridging triangles, distance and same neighbourhood: *θ*_*T*_ = -0.2, *θ*_*AT*_ = 1.0, *θ*_*LT*_ = 1.1, *θ*_*BT*_ = -2.2, *θ*_*D*_ =-2.6, *θ*_*S*_ =0.9; redundant ties 2% (dotted lines represent redundant ties)

Networks coherent with Burt’s theory might be generated tuning the parameters of local and bridging triangles and those of the elementary configurations whose interactions define them. Fig. 6a and b show that increasing the parameter *θ*_*LT*_ of the local triangles and assigning negative values to the parameter *θ*_*BT*_ of bridging triangles is not enough to obtain structurally efficient networks. In fact, the resulting structure of the network is composed of several components, corresponding to the neighbourhoods, which might be weakly connected according to the size of the parameters. Again, the percentage of redundant ties in the network is reduced (9% and 16% respectively) only because the choice of the parameters privileges the formation of ties within neighbourhoods.

To reproduce structurally efficient networks the parameters of triangles, alternating-k-triangles, distance and the same neighbourhood must also be tuned (Mills *et al.* 2013; Peeples and Haas 2013; Borck *et al.* 2015). In order to obtain networks that are more connected and characterized by clustering of ties and triangles, the value of the parameter of the triangles was decreased, whereas the values of the parameters of alternating-k-stars and distance were increased. Fig. 6c and d show two examples of networks drawn from ERGMs specified by the choice of the parameters described above. The sampled networks are characterized by one strong component, where each site can potentially have direct or indirect contacts with all the other sites. This differentiates these networks from those in Figs. 5b, 6a and b.

Fig. 6c and d differ in the size of *θ*_*LT*_ and *θ*_*BT*_. The two pictures show that increasing *θ*_*LT*_ and *θ*_*BT*_ yield networks characterized by a well-developed system of contacts within the close range and the presence of non-redundant outside contacts controlled by broker sites. Compared to the network in Fig. 4b, the percentage of redundant ties is lower (20% and 2% respectively). This is determined by the value of *θ*_*LT*_ and *θ*_*BT*_: the more extreme the values, the lower the percentage of redundant ties.

**Fig. 7:**
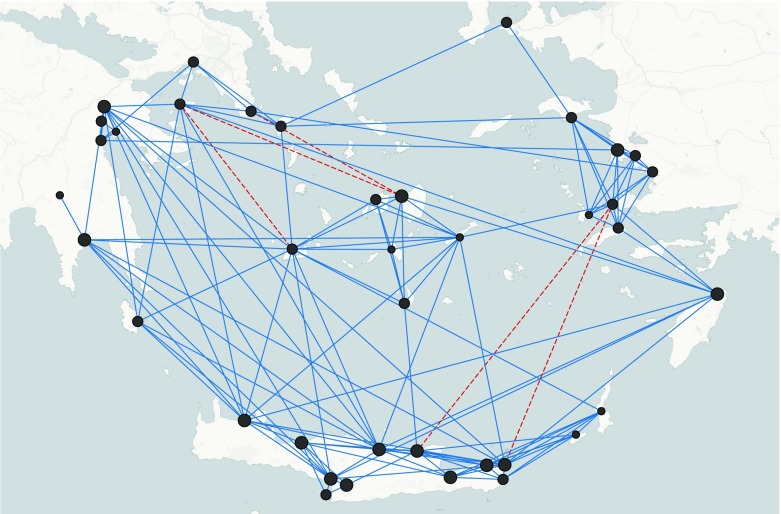
An example of a network reconstructed applying Burt’s theory and assuming that larger sites are playing the role of brokers in the contact networks. Parameters values: *θ*_*T*_ = -0.2, *θ*_*AT*_ = 1.0, *θ*_*LT*_ = 1.3, *θ*_*BT*_ = -2.4, *θ*_*D*_ = -2.6, *θ*_*S*_ = 0.9, *θ*_*A medium*_ = 0.75, *θ*_*A large*_ = 1.2; redundant ties 4% (dotted lines represent redundant ties)

Finally, structurally efficient networks where large sites play the role of brokers can be reconstructed tuning the parameter related to the effect of the size of sites on the formation of ties. Fig. 7 provides an example of a network based on this assumption.

## Discussion

In this paper we considered the issue of reconstructing past networks when fragmentary archaeological data does not provide enough information to get anywhere near a complete picture of network in the past. In this situation, the best we can do is to infer links using a model-based approach. This procedure requires the formulation of assumptions regulating the occurrence of ties in the past and the development of models determining which ties were more likely to exist.

The models used so far assume that presence and strength of ties depend only on factors which are exogenous to the network so that the only determinants of ties are characteristics of sites, such as their geographical location and size. Consequently, these models are not able to infer missing ties based on tie dependence assumptions and they limit the number of possible network scenarios. In this paper, we provided an example of assumptions that account for dyadic dependence and we showed how statistical network models may be used to reconstruct archaeological networks. In particular, we considered Burt’s theory based on closure and brokerage; we used it to define structurally efficient networks; and, by way of an example, we illustrated how exponential random graph models (ERGMs) can be applied to reconstruct structurally efficient networks.

On the one hand, the employment of ERGMs to infer ties in the past takes a step back from gravity models and ariadne. While ERGMs for binary relations are well-developed for both directed and undirected relations, extensions to the analysis of valued networks are still under investigation. Therefore, using ERGMs to reconstruct archaeological networks is currently limited to binary relations. Moreover, compared with ariadne, ERGMs do not account for the interdependence between strength of ties and site sizes. On the other hand, ERGMs takes a step forward from the previous models since they allow reconstructing networks using assumptions based on tie dependence and open up a variety of scenarios that cannot always be investigated by previous models. Furthermore, ERGMs enable us to reconstruct networks based on and controlling for several archaeological assumptions at the same time.

So far, different propositions describing the formation of ties in the past have always lead to the definition of new models. In fact, the mathematical formulation of MDN, PPA, GMs and ariadne is entirely determined by the assumptions on which they rest. However, archaeological propositions concerning tie formation often depend on time period, geographical area and the type of relations under investigation. Therefore, the development of a more flexible model capable of dealing with diverse archaeological contexts is desirable.

The example in Section 4 illustrated that ERGMs might be this flexible model. In fact, in ERGMs, different assumptions require only a different specification of the model, *i.e.* the choice of the local configurations and the tuning of the corresponding parameters. For instance, adding to Burt's theory the assumption that larger sites play the role of the brokers required changing the values of the site activity parameters.

Furthermore, particular specifications of ERGMs correspond to some of the previous models. MDN and PPA are non-stochastic counterparts of spatial Bernoulli random graphs (Butts 2002), a special type of ERGMs assuming that ties are independent and the probability of having a contact between any two sites is solely determined by the distance between them. When this probability is determined by the step functions in (1) and (2), the spatial Bernoulli random graphs correspond to MDN and PPA respectively. In its simple and binary formulation, also the gravity model can be written in an ERGM form. The corresponding ERGM is specified by the following configurations: edges, site activity and distance.

It follows that the reconstruction of archaeological networks using ERGMs turns into a model specification problem rather than the formulation of new models. This suggests that the “translation” of assumptions into network configurations is an important step in network reconstruction. Thus, it is fundamental to provide a framework where the main archaeological assumptions are matched to the corresponding local configurations. For instance, propositions stating that sites occupying a special place or having a special function have a tendency to be connected with a high number of sites can be incorporated into the model using statistics similar to the size site statistic. The description of the general framework is a topic of future work and is motivated by current research in the Caribbean.

Throughout this paper we assumed that archaeological networks are composed of settlements, whose existence is proved by the finding of sites, and ties representing contacts between them. We made this assumption because most of the applications of the previous models concern this type of settings, as attested by the case studies mentioned in Section 2. However, ERGMs can, in principle, be used to reconstruct networks on a lower (*e.g.* households) or a larger scale (*i.e.* cities, islands or region). In general, the scale of the network is determined by the scale at which the tie formation propositions are formulated and the possibility of translating these assumptions into local configurations. For instance, Burt’s theory can be applied to a regional level by assuming that there was a well-developed system of contacts within the close-range region and the presence of “gateways regions” connecting regions that otherwise would have been disconnected.

Finally, it must be noted that in the proposed approach ERGMs are used to reconstruct the global structure of past networks from micro-mechanisms defined on the tie-level. Although the generated networks can be thought of as the results of a series of tie changes determined by the tendency/preference of nodes for forming certain ties, the outcome is essentially a static network. Therefore, research questions concerning the evolution of the network over-time or the diffusion of practices and innovations, can be better addressed using agent-based or dynamic network models rather than ERGMs.
